# Multidrug resistance of uropathogenic *Escherichia coli* strains isolated from diabetic patients

**DOI:** 10.1186/1471-2334-14-S7-P44

**Published:** 2014-10-15

**Authors:** Oana Mariana Ionete, Carmen Avrămescu, Maria Bălăşoiu, Carmen Dobjanschi

**Affiliations:** 1 University of Medicine and Pharmacy Craiova, Romania; 2 Carol Davila University of Medicine and Pharmacy, Bucharest, Romania

## Background

Urinary tract infections (UTI) in diabetic patients are due to certain pathogenic microorganisms, the most common being *Escherichia coli*. There can also be selected multiple bacterial strains resistant to antibiotics. There are several risk factors predisposing to infection in these patients, especially if glycemic control is poor.

## Methods

This study conducted for one year included patients with type 1 and 2 diabetes hospitalized in the clinic for diabetes, nutrition and metabolic diseases in the Emergency County Hospital of Craiova, with or without symptoms of UTI. To make a distinction between contamination and true infection is important which method is used for this purpose. We used the midstream urine collection, especially in females. From these samples collected we have determined germs involved in UTI and have tested resistance to antibiotics of *Escherichia coli* strains isolated.

## Results

Analyzing the degree of multidrug resistance of *Escherichia coli* strains isolated from urine samples, related to ciprofloxacin resistance, we observed that of the 97 strains studied from diabetic patients hospitalized in the clinic for diabetes, nutrition and metabolic diseases, 11 strains showed different degrees of multidrug resistance representing a percentage of 11.34. Multidrug resistance includes strains resistant to 2 to 7 antibiotics. Among the 11 strains studied, 9 had intermediate susceptibility to 2 to 6 antibiotics.

## Conclusion

The number of multidrug resistant *Escherichia coli* strains compared to ciprofloxacin resistance was relatively low. Inappropriate use of antibiotics creates multidrug resistance. Meropenem remains a reserve antibiotic for multidrug resistant *Escherichia coli* strains, because all tested strains were susceptible. Drug resistance surveillance must be done periodically and it can be informative for appropriate management of antimicrobial resistance and for adequate and effective treatment.

